# Small intestinal press‐through package perforation

**DOI:** 10.1002/jgf2.519

**Published:** 2022-01-17

**Authors:** Toshimasa Yamaguchi

**Affiliations:** ^1^ 13877 Primary Care and Advanced Triage Section Osaka City General Hospital Osaka Japan

**Keywords:** foreign body ingestion, imaging, intestinal perforation, peritonitis, press‐through package, small intestine

## Abstract

A 75‐year‐old man had worsened abdominal pain. Contrast‐enhanced computed tomography revealed wall thickness in the terminal ileum with peritonitis. A round, high‐density object surrounded by a low‐density area, which is a characteristic computed tomography finding for a tablet in a press‐through package, was observed in the terminal ileum.

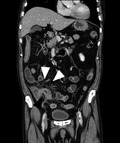

A 75‐year‐old man with type 2 diabetes mellitus and hypertension presented with a 5‐day history of worsened abdominal pain. His body temperature, blood pressure, pulse rate, and respiratory rate was 38.2°C, 146/80 mmHg, 92 beats/min, and 24 breaths/min, respectively. We also observed tenderness in the right lower abdomen, along with a muscular defense and attenuated bowel sounds. Laboratory tests revealed a white blood cell count of 8.71 × 10^9^/L (3.17–8.40) with 84.2% neutrophils and a high C‐reactive protein level of 161.5 mg/L (reference < 3.0). Contrast‐enhanced computed tomography revealed significant wall thickness in the terminal ileum, along with free air and dirty fat signs. A round, high‐density object surrounded by a low‐density area, which is a characteristic computed tomography finding for a tablet in a press‐through package (PTP), was observed in the terminal ileum (Figure 1). The patient was diagnosed with acute peritonitis and terminal ileum perforation caused by the PTP. Emergency laparotomy revealed a perforation in the terminal ileum and abscess formation in the thickened wall around the perforation. Subsequently, the patient underwent ileocecal resection, and the PTP was found in the resected ileum (Figure 2). The postoperative course was uneventful.

**FIGURE 1:**
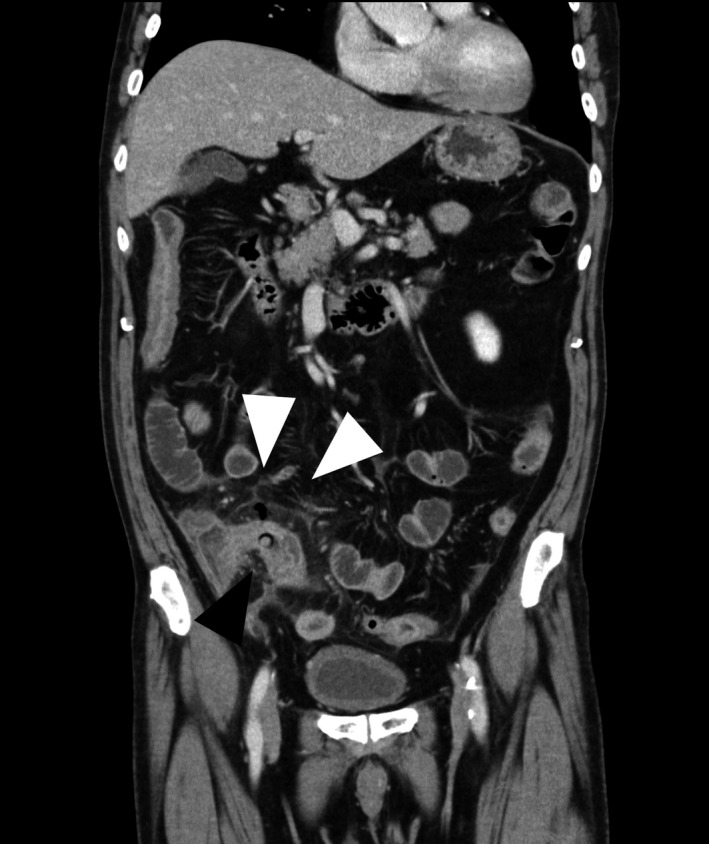
Coronal plain computed tomography demonstrating wall thickening, free air, and dirty fat signs (white arrowheads) in the terminal ileum, and a round, high‐density object, suspected to be a tablet in a press‐through package, surrounded by a low‐density area (black arrowhead)

**FIGURE 2:**
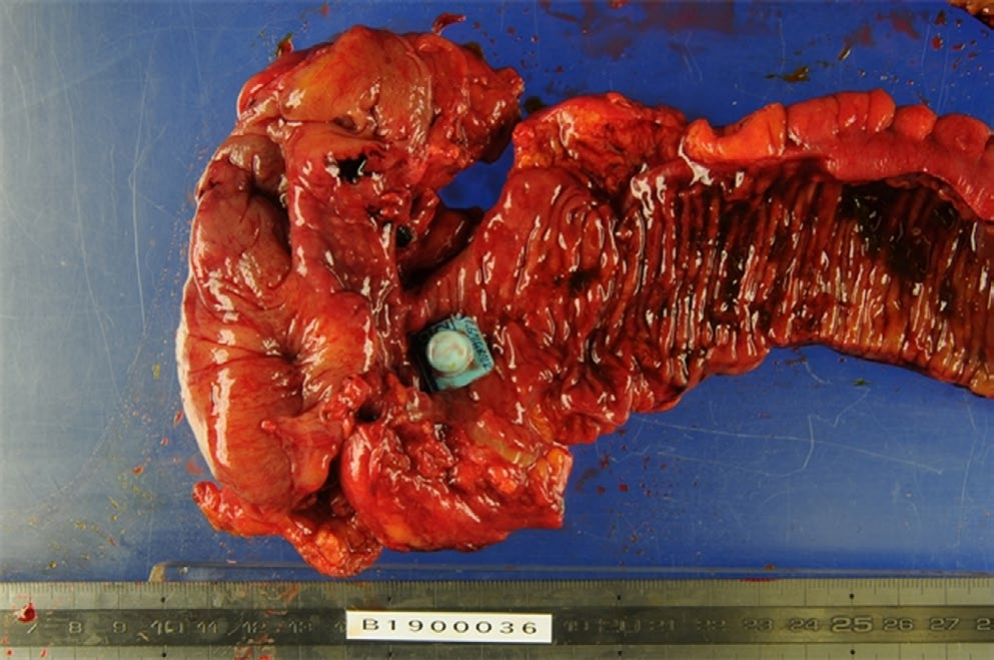
A small square press‐through package with sharp edges in the terminal ileum specimen

Approximately 75% of swallowed foreign bodies are reportedly spontaneously expelled, and approximately 1% cause intestinal perforation.^1^ Perforation owing to foreign bodies is commonly observed in areas where the intestinal anatomy is narrow or at an angle, such as at the cricopharyngeus, lower esophagus, or the ileocaecal and rectosigmoid junctions.^1^, ^2^

Currently, in Japan, the perforation line for PTPs is made in only one direction, such that they cannot be divided into small pieces to prevent accidental PTP ingestion. However, older adults sometimes divide PTPs into single‐dose pieces using scissors to easily identify the right amount of medicine. A PTP cut into a small square has sharp edges that may cause intestinal perforation. Accidental ingestion of PTPs is common among the elderly, and it is particularly difficult to obtain accurate medical history from patients with psychological diseases or dementia. Further, some patients may initially ignore or be unaware of accidental PTP ingestion.^3^

In this case, the patient had abdominal pain with a muscular defense. This condition could indicate an acute abdomen, which demands appropriate diagnosis and immediate decision on the need for emergency surgical treatment. Therefore, considering the accuracy and sensitivity of computed tomography on diagnosing acute abdomen, computed tomography was performed first on this patient without a plain abdominal radiograph, resulting in the definitive diagnosis of intestinal PTP perforation. Plain radiographs may also sometimes identify foreign bodies in the intestinal tract or abdominal cavity. Radiographs may be considered more beneficial to patients with accidental ingestion of PTP than computed tomography based on the radiation exposure or cost‐effectiveness if intestinal PTP perforation could be identified by plain radiographs. However, PTPs are difficult to detect on plain radiographs owing to their radiolucency. Computed tomography is sensitive to swallowed foreign bodies, including PTPs.^4^ Although the number of patients was small, the sensitivity of plain radiograph for accidental PTP ingestion has been reported to be 0%.^4^ However, it has been reported that PTPs cannot always be identified on computed tomography.^5^ Moreover, small intestine perforation is considered to be challenging to diagnose since free air is found in only approximately 50% of the patients with small intestine perforation.^5^ Therefore, the diagnosis of abdominal pain of unknown origin requires comprehensive evaluation, including imaging and evaluation of the clinical course and social background. Accidental PTP ingestion should be considered in those with underlying medical issues, particularly in elderly patients with polypharmacy.

## CONFLICT OF INTERESTS

The authors have stated explicitly that there are no conflicts of interest in connection with this article.

## INFORMED CONSENT

Patient consent for publication: Obtained.
